# AutoPrognosis 2.0: Democratizing diagnostic and prognostic modeling in healthcare with automated machine learning

**DOI:** 10.1371/journal.pdig.0000276

**Published:** 2023-06-22

**Authors:** Fergus Imrie, Bogdan Cebere, Eoin F. McKinney, Mihaela van der Schaar

**Affiliations:** 1 Department of Electrical and Computer Engineering, University of California, Los Angeles, California, United States of America; 2 Department of Applied Mathematics and Theoretical Physics, University of Cambridge, Cambridge, United Kingdom; 3 Department of Medicine, University of Cambridge, Cambridge, United Kingdom; 4 The Alan Turing Institute, London, United Kingdom; CSL Behring / Swiss Institute for Translational and Entrepreneurial Medicine (SITEM), SWITZERLAND

## Abstract

Diagnostic and prognostic models are increasingly important in medicine and inform many clinical decisions. Recently, machine learning approaches have shown improvement over conventional modeling techniques by better capturing complex interactions between patient covariates in a data-driven manner. However, the use of machine learning introduces technical and practical challenges that have thus far restricted widespread adoption of such techniques in clinical settings. To address these challenges and empower healthcare professionals, we present an open-source machine learning framework, AutoPrognosis 2.0, to facilitate the development of diagnostic and prognostic models. AutoPrognosis leverages state-of-the-art advances in automated machine learning to develop optimized machine learning pipelines, incorporates model explainability tools, and enables deployment of clinical demonstrators, *without* requiring significant technical expertise. To demonstrate AutoPrognosis 2.0, we provide an illustrative application where we construct a prognostic risk score for diabetes using the UK Biobank, a prospective study of 502,467 individuals. The models produced by our automated framework achieve greater discrimination for diabetes than expert clinical risk scores. We have implemented our risk score as a web-based decision support tool, which can be publicly accessed by patients and clinicians. By open-sourcing our framework as a tool for the community, we aim to provide clinicians and other medical practitioners with an accessible resource to develop new risk scores, personalized diagnostics, and prognostics using machine learning techniques.

**Software**: https://github.com/vanderschaarlab/AutoPrognosis

## Author summary

Previous studies have reported promising applications of machine learning (ML) approaches in healthcare. However, there remain significant challenges to using ML for diagnostic and prognostic modeling, particularly for non-ML experts, that currently prevent broader adoption of these approaches. We developed an open-source tool, AutoPrognosis 2.0, to address these challenges and make modern statistical and machine learning methods available to expert and non-expert ML users. AutoPrognosis configures and optimizes ML pipelines using automated machine learning to develop powerful predictive models, while also providing interpretability methods to allow users to understand and debug these models. This study illustrates the application of AutoPrognosis to diabetes risk prediction using data from UK Biobank. The risk score developed using AutoPrognosis outperforms existing risk scores and has been implemented as a web-based decision support tool that can be publicly accessed by patients and clinicians. This study suggests that AutoPrognosis 2.0 can be used by healthcare experts to create new clinical tools and predictive pipelines across various clinical outcomes, employing advanced machine learning techniques.

## Introduction

Machine learning (ML) systems have the potential to revolutionize medicine and become core clinical tools [1]. However, there are a diverse set of challenges that must be overcome prior to routine and widespread ML adoption [2, 3]. In particular, there are substantial technical challenges in developing, understanding, and deploying ML systems which currently render them largely inaccessible for medical practitioners [3–6].

In an attempt to address this, we previously developed AutoPrognosis, an automated machine learning (AutoML) framework that optimizes predictive pipelines [7]. AutoML aims to automate various aspects of the machine learning process. Initial AutoML approaches performed Neural Architecture Search [8] or hyperparameter optimization [9]. More recently, prior work has focused on both selecting the best algorithm and optimizing its hyperparameters from a pre-defined set, known as the combined algorithm selection and hyperparameter optimization (CASH) problem [10, 11]. However, limited work focused on optimizing full ML *pipelines*, and almost all existing frameworks could only handle complete data (i.e. without missing values) and did not construct model ensembles. The initial version of AutoPrognosis [7] incorporated these components in an efficient manner, employing a novel Bayesian Optimization procedure using structured kernels to solve the pipeline selection and configuration problem (PSCP). Our framework has been since applied to derive prognostic models for cardiovascular disease [12], cystic fibrosis [13], and breast cancer [14], among a number of other indications [15–21]. However, our initial approach had significant limitations from both algorithmic and usability perspectives. Perhaps most significantly, it was limited to classification, did not include interpretability methods, and did not readily allow models to be shared.

Consequently, in this work, we describe AutoPrognosis 2.0, a framework that addresses several major obstacles limiting the development, interpretation, and deployment of ML methods in medicine. To the best of our knowledge, this is the first approach that can simultaneously: (1) solve classification, regression, and time-to-event problems; (2) optimize ML pipelines, determine the most appropriate models, and automatically tune hyperparameters; (3) identify key variables and novel risk factors, enabling clinicians to select different numbers of variables and understand the value of information; (4) provide a diverse range of model explanations, including feature-based, example-based, and closed-form risk equations; and (5) produce web-based applications, allowing models to be readily shared with the clinical community.

After describing AutoPrognosis 2.0, we outline major challenges facing clinical development and translation of diagnostic and prognostic modeling, and detail how AutoPrognosis addresses each challenge. Finally, we demonstrate the application of AutoPrognosis 2.0 in an illustrative scenario: prognostic risk prediction of diabetes using a cohort of 502,467 individuals from UK Biobank. However, we emphasize that AutoPrognosis can be applied to construct diagnostic and prognostic models for *any* disease or clinical outcome, and is explicitly designed to make model building accessible to both experts and non-ML experts. We have open-sourced AutoPrognosis 2.0 as a tool for the community, allowing model developers of all levels of expertise to robustly and reproducibly develop optimized personalized diagnostics, prognostics, and risk scores using modern machine learning techniques.

## Methods: AutoPrognosis 2.0

AutoPrognosis 2.0 is an algorithmic framework and software package that allows healthcare professionals to leverage ML to develop diagnostic and prognostic models. Our framework employs automated machine learning [11] to tackle the challenges faced by clinical users. By automating the optimization of ML pipelines involving data processing, model development, and model training, we reduce the burden on technical experts and turn deriving ML models from an art to a science, democratizing machine learning and opening the field to non-ML domain experts, such as clinicians. We believe that AutoPrognosis 2.0 represents a step-change in algorithmic and software capabilities and can unlock the potential of ML in healthcare for clinical researchers *without* the requirement for extensive technical capabilities.

AutoPrognosis 2.0 empowers users with the following capabilities:

1. Build highly performant ML pipelines for classification, regression, and time-to-event analysis, optimized specifically for the data at hand.
2. Understand when ML provides benefits over traditional regression models, and thus when ML is valuable.
3. Enable principled selection of variables and allow users to understand the value of information.
4. Explain and debug how ML models issue predictions using diverse interpretability methods.
5. Update systems whenever the available data changes to ensure the best possible clinical models.
6. Provide confidence in the reproducibility of models.

### Overview

After a clinician has determined an appropriate cohort of patients and an outcome of interest, the AutoPrognosis framework handles all steps in the computational pipeline: missing data imputation, feature processing, model selection and fitting, model interpretability or explanations, and production of clinical demonstrators. Together, we believe AutoPrognosis significantly reduces the technical expertise necessary to derive powerful prognostic models, empowering clinical users and democratizing machine learning in healthcare.

**Fig 1:**
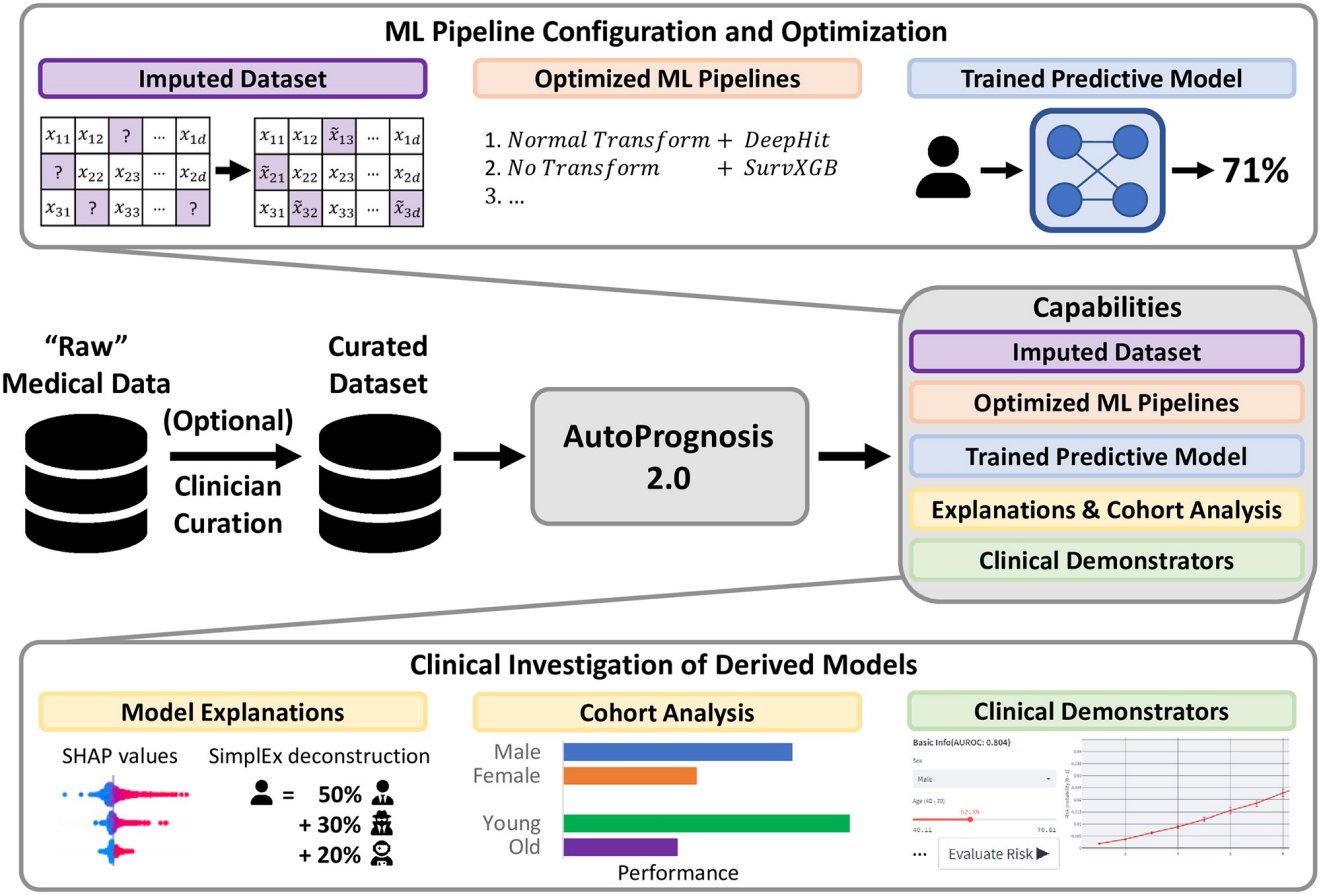
Overview of the AutoPrognosis 2.0 framework. AutoPrognosis takes as input a medical dataset and provides an imputed dataset, a report detailing the optimized machine learning pipelines, a diagnostic or prognostic model, explanations, and a web-based interface for clinicians to interact with and use the derived model.

AutoPrognosis is provided as an open-source package at https://github.com/vanderschaarlab/AutoPrognosis and can be readily installed with PyPI (https://pypi.org/project/autoprognosis/). AutoPrognosis is primarily intended as a Python package, but we also provide bindings for R users. AutoPrognosis 2.0 requires only basic familiarity with either language for successful deployment. Note that, as for any computational approach, care must be taken when preparing data for use with AutoPrognosis. However, while the package cannot prevent input of inappropriate data (as no package can), it does ensure the selection of appropriate and optimal methods and hyperparameters for each step in the pipeline outlined in Table 1. An overview of AutoPrognosis 2.0 is provided in Fig 1. Below, we provide a summary of each of the core components of AutoPrognosis.

**Table 1:** List of algorithms currently included in AutoPrognosis 2.0. Algorithms grouped by pipeline stage. Numbers in brackets correspond to the number of hyperparameters optimized over by AutoPrognosis. AutoPrognosis is readily extendable to additional methods, algorithms, and hyperparameters.

Pipeline Stage	Algorithm (No. Hyperparameters Optimized by AutoPrognosis)				
**Imputation**	HyperImpute (M)ICE (0)	Mean (0) SoftImpute (2)	Median (0) EM (1)	Most-Frequent (0) Sinkhorn (6)	MissForest (2) None (0)
**Dimensionality Reduction**	Fast ICA (1)	Feat. Agg. (1)	Gauss. Rand. Proj. (1)	PCA (1)	Var. Thresh. (0)
**Feature Scaling**	L2 Norm. (0) Unif. Trans. (0)	Max (0) None (0)	MinMax (0)	Normal Trans. (0)	Quant. Trans. (0)
**Classification**	ADABoost (3) ExtraTree (1) LDA (0) Neural Net. (6) TabNet (8)	Bagging (4) Gauss. NB (0) Light GBM (6) Perceptron (2) XGBoost (11)	Bernoulli NB (1) Grad. Boost. (3) Linear SVM (1) QDA (0)	CatBoost (2) Hist. Grad. Boost. (2) Log. Reg. (4) Random Forest (5)	Decision Tree (1) KNN (4) Multi. NB (1) Ridge Class. (1)
**Regression**	Bayesian RR (1) TabNet (8)	CatBoost (2) XGBoost (2)	Linear (0)	MLP (0)	Neural Net. (6)
**Survival Analysis**	Cox PH (2) Surv. XGB (4)	CoxNet (6) Weibull AFT (2)	DeepHit (7)	LogLogistic AFT (1)	LogNorm. AFT (2)
**Interpretability**	INVASE SimplEx	KernelSHAP Symb. Pursuit	LIME	Effect Size	Shap Permutation

### Missing data imputation

Medical datasets are often incomplete; however, most models require complete data as input, thus imputation is a necessary first step. There are many different imputation methods available, ranging from traditional statistical approaches such as mean imputation to well-known alternatives such as MICE [22] and MissForest [23]. We include eight common imputation algorithms in AutoPrognosis for users to select if they desire a specific imputation method.

In addition, we also include a state-of-the-art AutoML approach for imputation, HyperImpute [24]. HyperImpute is a generalized iterative imputation algorithm that automatically configures feature-wise imputation models. HyperImpute inherits the usual properties of classical iterative imputation algorithms [22, 25, 26] while benefiting from an automated model selection and hyperparameter optimization procedure that allows the most appropriate model to be chosen for each feature. HyperImpute optimizes over five classes of model, with a total of 29 configurable hyperparameters. For additional details, we refer to the recent technical report detailing HyperImpute [24]. HyperImpute is the recommended imputation strategy in AutoPrognosis unless a specific method is preferred by the user. Alternatively, the imputation step can be jointly optimized as part of a larger pipeline.

### Developing optimized ML pipelines

After imputation, we construct ML pipelines consisting of feature processing, model selection, and model fitting. Given an objective function, these steps are jointly optimized using AutoML. There are several possible choices for the pipeline search algorithm, such as Bayesian optimization [7, 27] or bandit-based approaches [28]. A key difference in this work is the extension of such approaches beyond hyperparameter optimization, the typical use of AutoML, to accommodate more general configuration spaces that encompass ML pipelines. AutoPrognosis is flexible to the choice of AutoML search algorithm and can be extended as new approaches are developed. Currently, our default approach is based on Bayesian optimization but we have also included an extension of Hyperband [28]. In Table 1, we provide a list of the algorithms currently implemented in AutoPrognosis 2.0, together with the number of hyperparameters optimized over for each method. We emphasize the extendability of our approach to new methods, algorithms, and hyperparameters.

#### Feature processing

While imputation ensures data is complete, preprocessing datasets is a common requirement for many ML estimators. In particular, feature scaling to normalize the range or the shape of features can significantly affect performance [29]. AutoPrognosis can optimize over five dimensionality reduction and six feature scaling algorithms.

#### Model selection and fitting

Next, a model and hyperparameters must be selected. This is a key step as suboptimal choice of model or hyperparameters can significantly affect the performance of the resulting ML system. AutoPrognosis contains 22 classification algorithms, seven regression algorithms, and seven methods for survival analysis. Together with a range of hyperparameters, this defines a broad algorithmic search space. While navigating this space manually by hand is extremely challenging, AutoPrognosis learns relationships between different settings to efficiently arrive at an optimized solution. Finally, AutoPrognosis combines the best-performing models into a single ensemble. AutoPrognosis can construct ensembles that are weighted combinations of the best-performing models or stacking ensembles, where a meta-model is placed on top of the underlying models. For the illustrative application included in this paper, we used weighted ensembles.

### Model explanations

Predictive models alone are not sufficient and a deeper understanding is required to engender model trust from both clinical users [5] and regulatory bodies [30–32]. Consequently, AutoPrognosis contains a suite of methods for explaining ML models. We have included feature-based interpretability methods, such as SHAP [33], that allow us to understand the importance of individual features, as well as an example-based interpretability method, SimplEx [34], that explains the model output for a particular sample with examples of similar instances, similar to case-based reasoning. Furthermore, sometimes outputs of a specific form are required, such as explicit risk equations [32]. We have therefore included the ability to convert optimized models into transparent risk equations using symbolic regression [35].

### Demonstrators

In order for risk scores to be useful, they need to be readily available to clinical practitioners. To facilitate this, AutoPrognosis allows interactive demonstrators to be produced for clinical use. We build our clinical demonstrators on top of the open-source Streamlit package [36]. Compared to traditional solutions, these require almost no technical capabilities to set up, and the standardized nature simplifies adoption for end-users.

## Challenges in diagnostic and prognostic modeling

There are numerous obstacles to developing and deploying diagnostic and prognostic models that currently prevent healthcare professionals from capitalizing on recent algorithmic advances [1]. Our work seeks to empower clinicians, medical researchers, epidemiologists, and biostatisticians through an accessible, automated framework capable of identifying optimal solutions to all major obstacles limiting ML model building with minimal need for technical expertise. We begin by describing seven major challenges faced by these communities and how they are addressed by AutoPrognosis 2.0 (Table 2).

**Table 2:** Major challenges facing clinical development of diagnostic and prognostic models and how these are addressed by AutoPrognosis. See Challenges in diagnostic and prognostic modeling for more detail.

Challenge 1. Developing powerful ML pipelines
AutoPrognosis uses AutoML to automate pipeline configuration, performing missing value imputation, feature processing, model selection, and hyperparameter optimization.
Challenge 2. Understanding the value of ML and when it is necessary
AutoPrognosis compares a range of ML methods to traditional approaches and automatically identifies what approach is best.
Challenge 3. Determining the value of information
AutoPrognosis can quantify the value of including additional predictors, enabling systematic identification of optimal variables.
Challenge 4. Understanding and debugging ML models
AutoPrognosis incorporates seven state-of-the-art interpretability methods, allowing models to be understood and debugged as they are generated.
Challenge 5. Making ML models accessible and usable
AutoPrognosis provides a platform to share model outputs by automating the creation of web-based applications.
Challenge 6. Deciding when and if to update clinical models
AutoPrognosis can quantify the benefit of additional data or new predictive variables, and automatically determine the optimal system for the new dataset.
Challenge 7. Transparent reproducibility
AutoPrognosis provides a standardized, publicly available framework, facilitating reproducibility.

### Challenge 1. Developing powerful ML pipelines

Developing performant ML models remains complex and typically involves significant time and effort for both clinicians [37] and expert ML practitioners [38] alike. Indeed, some estimates suggest over 95% of work is expended on software technicals, leaving less than 5% for addressing the medical or scientific problem at hand [39]. This is further complicated by the myriad of choices that must be made when developing a new predictive model for diagnosis or prognosis, such as: what imputation strategy should be used; how should the data be preprocessed; what (ML) model is best suited for the specific task; what configuration of hyperparameters should be used. These decisions affect each other, thus cannot be made in isolation [38]; further, the optimal choices not only vary between applications, but also can change over time as more data is collected and clinical practice changes [40].

Few resources are available to help empirically define optimal computational pipelines. AutoPrognosis 2.0 addresses this by incorporating an AutoML approach within a standardized framework, automating the process of pipeline configuration. AutoPrognosis navigates a broad algorithmic search space in an efficient fashion, systematically performing missing value imputation, feature processing, model selection, and hyperparameter optimization in an unbiased manner without the need for human intervention or expert insight. This avoids arbitrary parameter selection and ensures standardization of pipelines, facilitating both reproducibility and optimized model performance. Critically, this democratizes the model building step, eliminating the requirement for expert ML knowledge and making cutting-edge methodology accessible to all, freeing healthcare domain experts to define and address the core clinical problems.

### Challenge 2. Understanding the value of ML and when it is necessary

Traditional approaches, such as linear regression and Cox proportional hazard models [41], are widely used and accepted across healthcare. Before replacing these established methods, it is vital to understand whether ML is valuable for a given problem and quantify the benefit of ML systems. Indeed, there is no “free lunch” and we should not expect ML to always outperform existing approaches [42]. Further, simple solutions can be desirable [43]. Several recent examples exist that present settings where comparatively “simple” approaches outperformed ML [44, 45].

AutoPrognosis 2.0 can be used to compare a range of ML methods to traditional approaches at minimal technical cost to the user. Furthermore, since these solutions are included in the algorithmic search space, AutoPrognosis will automatically identify whether such approaches are indeed best or if more complex ML models are required.

### Challenge 3. Determining the value of information

Selecting which variables to include in a predictive model is a critical aspect of model development that not only impacts model performance but also the ease of subsequent clinical use [46]. This is due to models with fewer features being easier to interpret and use in practice [47] but also since any feature used will need to be collected in an ongoing manner to use such systems. Thus, understanding the *value* of an individual variable and the information it provides is critical. Often, this is assessed by univariate statistical analysis or other selection methods such as forward selection or backward elimination [48]. AutoPrognosis 2.0 provides methods to test and quantify the value of including additional predictors, allowing systematic identification of optimal variables in an informed manner.

### Challenge 4. Understanding and debugging ML models

A predictive clinical model must be more than just accurate, it must be interpretable. Without a transparent understanding of *how* a model makes predictions it may act in unintended and undesirable ways, for example learning incorrect or aberrant features unique to the training data [49, 50]. In particular, model debugging can be used to check for shortcut learning [51], where the model learns spurious relationships in the provided data, or data leakage [52], which can lead to overly optimistic performance estimates. As seen in several machine learning applications in healthcare [50, 53, 54], shortcut learning can be a serious issue that must be avoided. Additionally, fairness and bias are two important considerations when developing any predictive model, particularly in healthcare [55], and existing societal biases in the data should not be reinforced by models [43]. While related to Challenge 1 (since a perfectly predictive model is both fair and unbiased), assessing fairness and bias, as well as understanding their origin, are key steps in model development and debugging. While interpretability does not guarantee that a model will be fair and unbiased, it creates the opportunity to assess these characteristics by probing how the model issues predictions.

The debugging step is critical for building model trust [5] and cannot be achieved without interpretation of the training features or cases that support model accuracy. It is clear that clinical deployment of an interpretable model is supported by the additional trust gained by understanding the model’s performance [56].

Furthermore, a clear understanding of computational models is now a requirement for deployment in healthcare systems globally: in the United States, the FDA demands “transparency about the function and modifications of medical devices” as a key safety aspect [30]; Article 22 of GDPR legislation in the EU requires that “meaningful information about the logic involved” be provided in certain circumstances [31]; and Article 13 (1) of the European Commision Proposal for the AI Act states “High-risk AI systems shall be …sufficiently transparent to enable users to interpret the system’s output”, among others. To achieve this transparency, interpretable outputs of a specific form can also be required. For example, the American Joint Committee on Cancer requires explicit risk equations [32].

The ‘black-box’ nature of many ML methods means that they remain inherently uninterpretable and require specialized methods to unravel the underlying rationale for predictions. In AutoPrognosis 2.0, we have incorporated seven state-of-the-art interpretability methods allowing researchers to understand and debug ML models as they are generated.

### Challenge 5. Making ML models accessible and usable

Predictive models need to be accessible to be used in clinical practice. This step often limits adoption, since bespoke deployment can result in significant costs and reliance on technical expertise. While full clinical deployment may require additional systems (e.g. due to regulatory requirements), a standardized, user-friendly solution to rapidly visualize and share models is also a necessary part of both debugging and confirming clinical acceptance. AutoPrognosis 2.0 provides a platform to share model outputs by automating the creation of web-based applications, allowing clinicians to explore predictions in diverse scenarios.

### Challenge 6. Deciding when and if to update clinical models

Over time, more data is collected, new variables are measured, and even clinical practice changes [57, 58]. For the former, existing clinical predictive models might benefit from additional data or features, while in the latter case, model performance may degrade [40]. However, deciding whether to update a clinical model is not a decision to be made lightly, since beyond model building, further regulatory approval might be necessary and the updated model will need to be redeployed. AutoPrognosis can help answer this difficult question by quantifying the benefit of additional data and new predictive variables, while also automatically determining the optimal system configurations for the new dataset, which may have changed.

### Challenge 7. Transparent reproducibility

Reproducibility is a fundamental requirement for the acceptance and adoption of any predictive model. While transparently reproducing a model’s output on a given dataset is conceptually simple, several factors can confound this necessary step. Serial data releases, code updates, and even inherent properties of ML algorithms (for example, stochastic descent methods can give different answers even when run repeatedly on the same data) can conspire to make ML model building less reproducible than it should be [59]. These issues demonstrably obstruct translation of clinical prediction and erode trust in ML approaches [60–62]. AutoPrognosis 2.0 addresses this major challenge by providing a standardized, publicly available framework to train predictive models, allowing straightforward demonstration of reproducibility on source data.

## Illustrative application: Diabetes risk prediction

In this section, we show how AutoPrognosis 2.0 can be applied to address the challenges described in Challenges in diagnostic and prognostic modeling. We demonstrate the application of AutoPrognosis 2.0 using an illustrative scenario: prognostic risk prediction of developing diabetes using a cohort of 502,467 individuals from UK Biobank. Our goal is *not* to develop the best model for diabetes risk prediction possible, but instead to exemplify how our tool can be used.

In our use scenario, we show that the model derived with AutoPrognosis outperforms risk models currently used in clinical practice and quantify the benefit of ML methods over Cox proportional hazard models. In addition, we show how the model interpretability components of AutoPrognosis can be used to understand the drivers of predictions and identify novel risk factors not incorporated into previous risk scores. Finally, we use AutoPrognosis to share the diabetes risk score as a web-based decision support tool that can be publicly accessed by patients and clinicians (https://autoprognosis-biobank-diabetes.streamlitapp.com/).

While we illustrate risk prediction of developing diabetes using a cohort from UK Biobank, AutoPrognosis can be applied to construct diagnostic and prognostic models for any disease or clinical outcome. Furthermore, AutoPrognosis is applicable to classification and regression tasks, in addition to survival analysis.

### Designing experiments

#### Selecting which dataset to use

AutoPrognosis can be used with data from many different origins, such as biobanks [12], registries [13, 14], and private hospital data [17]. Here, we use the UK Biobank due to its availability and popularity as a resource for healthcare researchers. UK Biobank enrolled half a million participants from 22 assessment centers across England, Wales, and Scotland between 2006 and 2010 [63], with follow-up data collected from hospital records [64]. From UK Biobank, we extracted a cohort of participants who were 40 years of age or older with no diagnosis or history of diabetes at baseline; the primary outcome was diagnosis of diabetes within a 10-year horizon. We selected diabetes as our outcome of interest due to its global prevalence and role as a risk factor for a multitude of other indications [65].

#### Selecting variables

Variables can be selected for inclusion in a study in a myriad of ways. Often, healthcare professionals will select a subset of exploratory features that are of particular interest to them. This could be due to supporting medical literature, to explore a hypothesis, or based on features included in existing risk scores. Alternatively, we can always choose to initially include all available variables. Here, we selected an initial set of 109 exploratory features based on their general clinical availability, discussions with clinicians, and features used by existing risk scores. Descriptive characteristics of the UK Biobank cohort are provided in S4 Table. Most variables had low levels of missingness (< 1%); however, some important variables had higher missingness rates (e.g. HbA1c: 6.8%). We purposefully selected almost an order of magnitude increase compared to existing risk scores to illustrate how AutoPrognosis can be used in such a scenario.

#### Selecting benchmarks

Often, existing risk scores will exist for the outcome of interest; this is certainly true for diabetes, where several risk scores that estimate the probability of developing diabetes are currently used in clinical practice. Therefore, we use the following as baseline risk scores:

- **ADA**: The American Diabetes Association (ADA) risk score [66] is a points-based score employing six features, namely age, sex, family history of diabetes, history of hypertension, obesity, and physical activity.
- **FINRISK**: A risk score for diabetes was derived from FINRISK, a large population survey in Finland, based on age, body mass index (BMI), waist circumference, history of antihypertensive drug treatment and high blood glucose, physical activity, and daily consumption of fruits, berries, or vegetables [67].
- **DiabetesUK**: The risk score from Diabetes UK uses seven features: gender, age, ethnicity, family history, waist size, BMI, and high blood pressure requiring treatment.
- **QDiabetes**: Finally, QDiabetes [68] consists of three separate models depending on the clinical information available and stage of risk screening. Model A uses 16 non-laboratory features that do not require a blood test and is intended primarily as an initial screening tool. Models B and C include the same variables as Model A together with fasting blood glucose and hemoglobin A1c (HbA1c), respectively, with the aim of refining risk assessment following a blood test.

In addition to the baseline risk scores, a comparison with traditional modeling approaches can be made using AutoPrognosis. We demonstrate this by fitting Cox proportional hazard (Cox PH) [41] models using the same features as each of the baseline risk scores. These models can be thought of as variants of the respective risk scores calibrated to the specific dataset.

### Results

Through the lens of our example (diabetes risk prediction), we demonstrate how AutoPrognosis 2.0 can be used to address the challenges of diagnostic and prognostic modeling introduced in Challenges in diagnostic and prognostic modeling.

#### Challenge 1. Developing powerful ML pipelines

We begin by using AutoPrognosis to derive a clinical risk score for diabetes. We evaluated the performance of the models using concordance index (C-index) to assess model discrimination, Brier score to assess calibration, and the area under the receiver-operating curve (AUROC) to assess prediction accuracy. We performed imputation five times and conducted 3-fold cross-validation for each of the imputed datasets.

As seen in Table 3, the risk score developed by AutoPrognosis significantly outperforms all baseline risk scores and Cox PH models (two-sample unpaired t-test between C-indices: p-value <0.001), achieving a C-index on the validation cohort of 0.888 (95% confidence interval: 0.881–0.895). This compares to 0.696 (0.681–0.711) for the ADA score, 0.728 (0.699–0.757) for FINRISK, 0.759 (0.746–0.772) for DiabetesUK, and 0.839 (0.818–0.860) for the best performing QDiabetes model (Model C). Cox PH models fit with the same risk factors as the clinical risk scores achieved improved performance (C-indices: 0.774, 0.786, 0.794, and 0.858, respectively), but exhibit lower performance than AutoPrognosis.

**Table 3:** Diabetes risk prediction results. The risk scores automatically derived by AutoPrognosis outperform the existing risk scores and Cox PH models retrained on the same features. Mean performance reported with 95% confidence interval.

Method	C-index ↑	Brier score ↓	AUROC ↑
ADA	0.696 ± 0.015	0.011 ± 0.000	0.697 ± 0.018
FINRISK	0.728 ± 0.029	0.019 ± 0.000	0.729 ± 0.020
DiabetesUK	0.759 ± 0.013	0.016 ± 0.000	0.759 ± 0.019
QDiabetes Model A	0.794 ± 0.022	0.008 ± 0.000	0.795 ± 0.017
QDiabetes Model B	0.788 ± 0.019	0.015 ± 0.000	0.788 ± 0.013
QDiabetes Model C	0.839 ± 0.021	0.005 ± 0.000	0.840 ± 0.010
Cox PH (ADA)	0.774 ± 0.027	0.002 ± 0.000	0.774 ± 0.020
Cox PH (FINRISK)	0.786 ± 0.023	0.002 ± 0.000	0.786 ± 0.026
Cox PH (DiabetesUK)	0.794 ± 0.023	0.002 ± 0.000	0.794 ± 0.022
Cox PH (QDiabetes C)	0.858 ± 0.007	0.002 ± 0.000	0.860 ± 0.018
**AutoPrognosis 2.0**	**0.888 ± 0.007**	**0.002 ± 0.000**	**0.888 ± 0.012**
AutoPrognosis (18 feat.)	0.870 ± 0.011	0.002 ± 0.000	0.867 ± 0.020

As an alternate way of understanding the clinical impact of our results, we performed decision curve analysis [69, 70]. Decision curve analysis assesses the clinical value of a predictor by calculating the clinical net benefit across a range of risk threshold probabilities, where the threshold probability is defined as the minimum probability of an event at which a decision-maker would take a given action. Net benefit is defined as the difference between the proportion of true positives and the proportion of false positives weighted by the odds of the selected threshold. Evaluating net benefit is recommended in the TRIPOD guidelines [71]. At any given threshold, the model with the higher net benefit is preferred.

**Fig 2:**
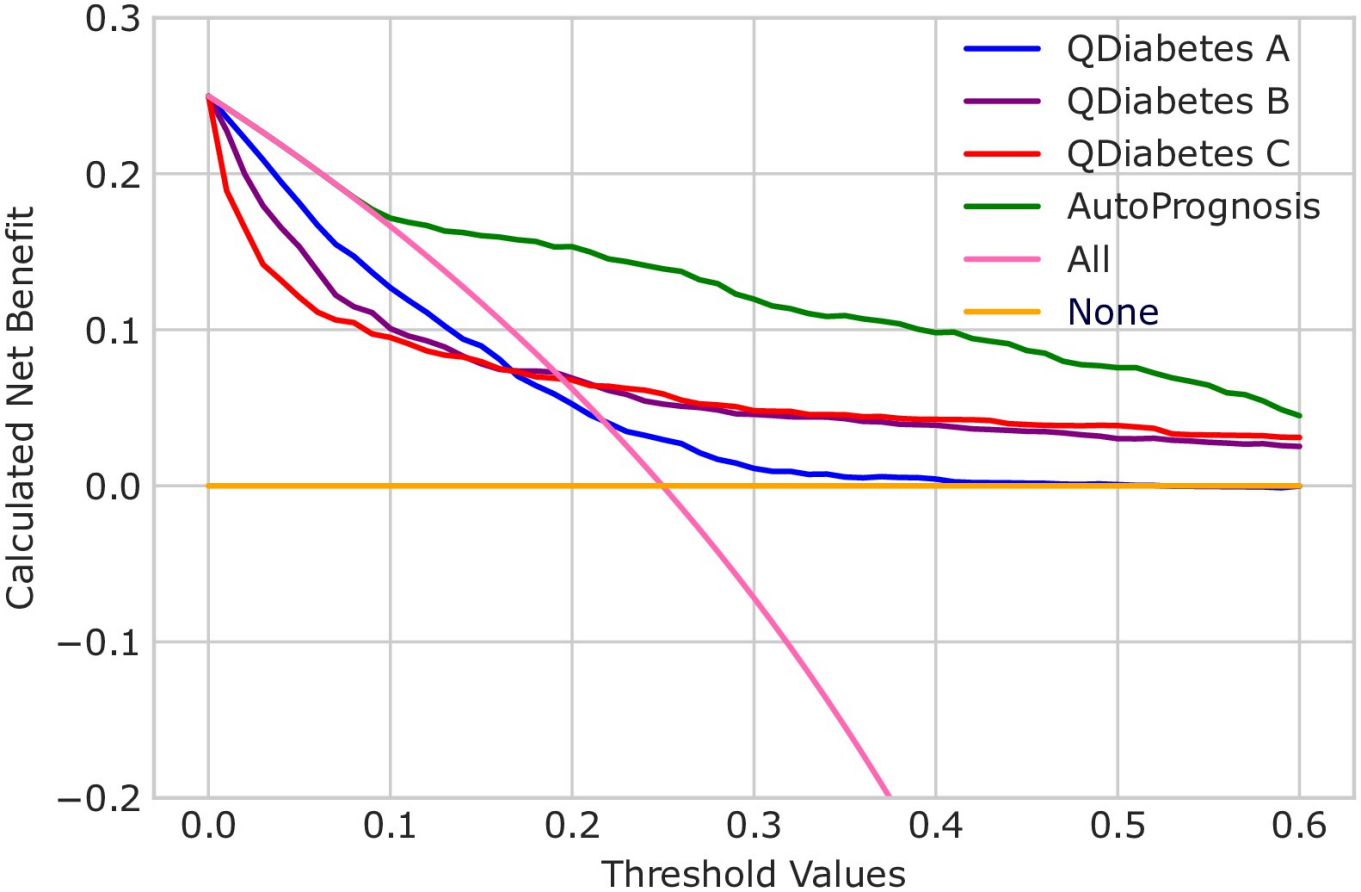
Decision curve analysis. AutoPrognosis exhibits higher net benefit at all decision thresholds compared to existing risk scores and baseline strategies.

We compared the predicted risk by AutoPrognosis with the QDiabetes models, the best performing of the existing clinical risk scores, as well as baseline strategies to assume all patients will develop diabetes (All) or that no-one will (None). Decision curve analysis further demonstrates the benefit of AutoPrognosis compared to existing risk scores for diabetes (Fig 2). At all decision thresholds, AutoPrognosis offers greater net benefit and is the only score to outperform “All” between the thresholds of 0.1 and 0.2, and the only model to perform similarly to “All” below a threshold of 0.1.

#### Challenge 2. Understanding when ML is necessary and its value

Table 3 demonstrates the benefit of AutoPrognosis compared to existing risk scores and Cox PH models retrained on the same features. We now directly compare AutoPrognosis to Cox PH models on the same training data to understand if ML is needed for this problem. In Table 4, we show the performance of AutoPrognosis and a Cox PH model using the full feature set considered. We see that while some of the benefit is due to the additional features, there remains value in the improved modeling approach, even for identical feature sets.

**Table 4:** Quantifying the value of ML. The risk score automatically derived by AutoPrognosis significantly outperforms a Cox PH model trained on the same features.

Method	C-index ↑
*All Variables*	
Cox PH	0.883 ± 0.010
AutoPrognosis	0.888 ± 0.007

#### Challenge 3. Determining the value of information

**Fig 3:**
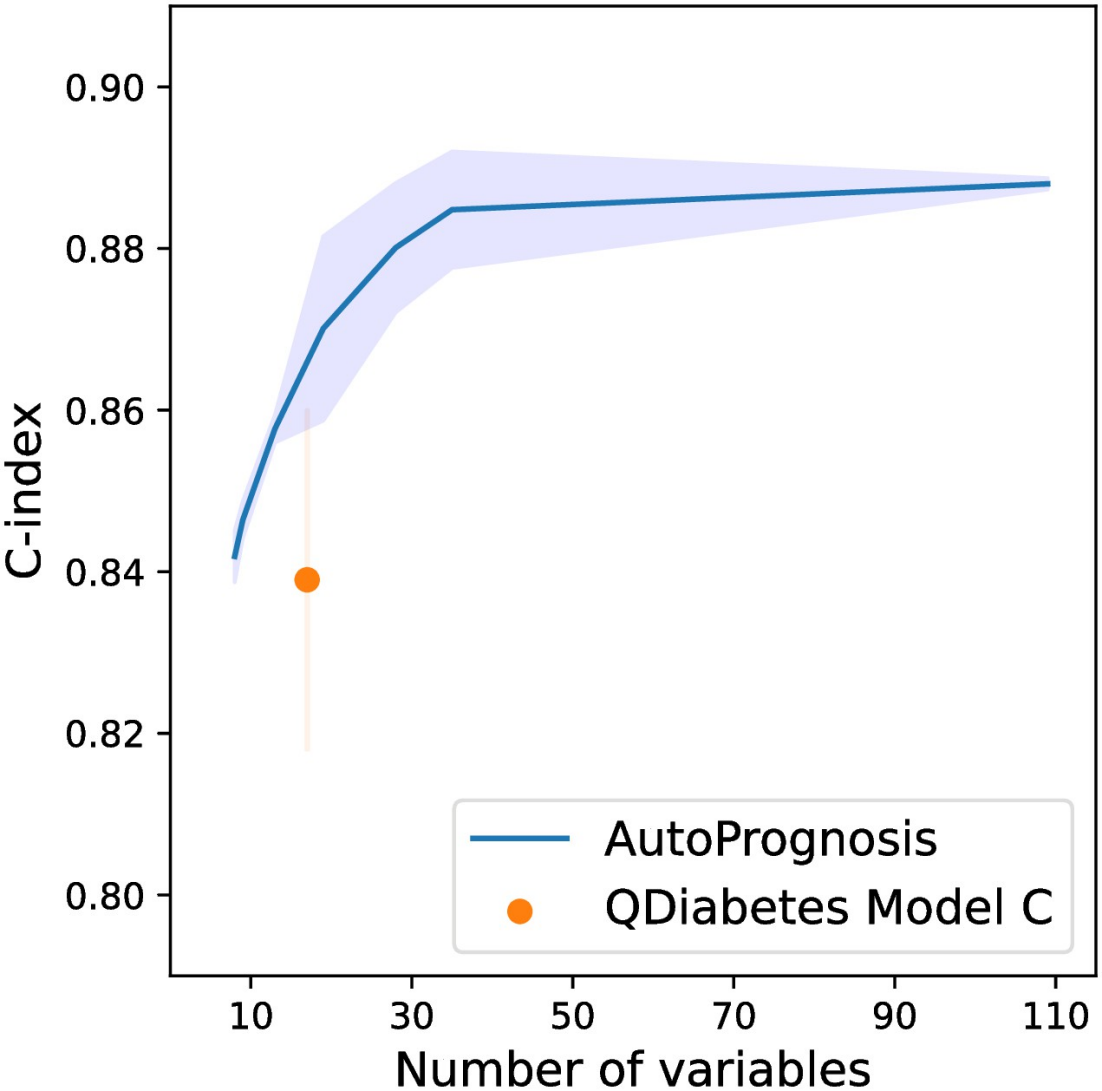
Value of information. We evaluate AutoPrognosis with different numbers of features, selected using effect size. Feature efficiency is compared to QDiabetes Model C, the best performing existing risk score. Note y-axis does not start at 0 nor end at 1.

Understanding the predictive power of variables is key and often there is a trade-off (e.g. cost or time) in clinical practice to acquiring additional variables. We evaluate AutoPrognosis using different subsets of features. We selected features using the magnitude of the effect size. We measure the distributional shift for an increase in predicted risk using Cohen’s D [72] and select features with effect sizes exceeding the thresholds {0.5, 0.6, 0.7, 0.8, 0.9, 1.0}. Even using only eight features (effect size: 1.0), AutoPrognosis slightly outperforms the best performing existing risk score, QDiabetes Model C, which employs 17 features (Fig 3). With a comparable number of features (18 features, effect size 0.7), AutoPrognosis displays significantly improved performance (Table 3). As the number of features increases, performance rapidly increases until 35 features are used (effect size: 0.5). After this point, while there is some gain from additional features, it could be considered marginal given the number of additional features employed. See S1 Table for the most important features using effect size.

#### Challenge 4. Understanding and debugging ML models

**Fig 4:**
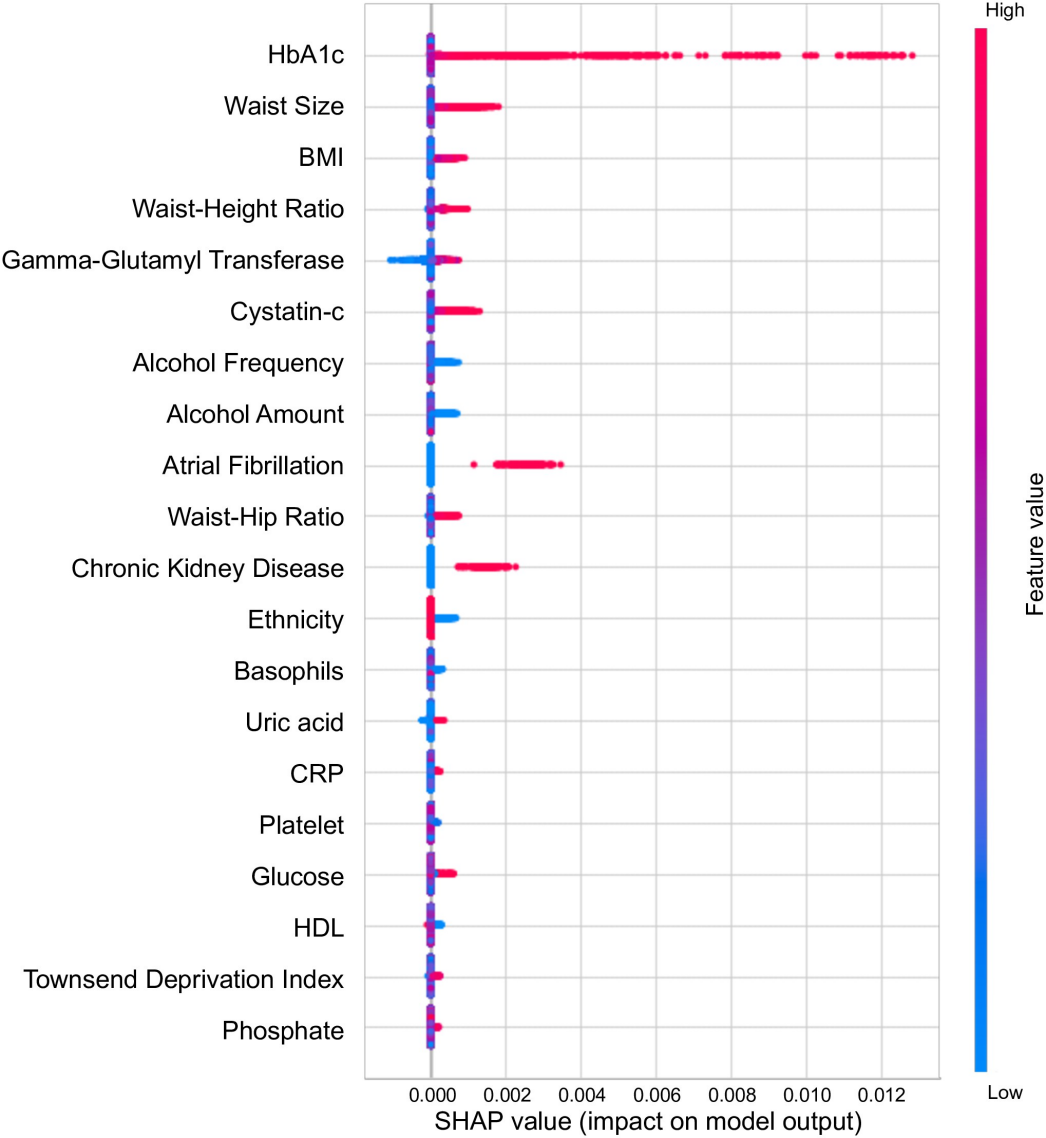
SHAP values for the most important features.

Highly predictive models alone are insufficient and it is necessary to understand which features are important. We demonstrate how the interpretability methods incorporated in AutoPrognosis 2.0 can be used to understand how ML models make predictions and debug their behavior. We begin by examining the SHAP values [33] to explain the key contributors to model performance. Fig 4 shows the top 20 features. Encouragingly, these features are largely consistent with clinical knowledge, providing evidence that the model is acting in a desirable manner. Several of the top risk factors, such as HbA1c, waist size, and body mass index, were also included in previous risk scores. However, a number of additional features, including both laboratory and non-laboratory tests, were deemed important. A number of these features have been shown to be risk factors for diabetes (e.g. gamma-glutamyl transferase [73]), but have not been incorporated into other risk scores. Of the existing risk factors, we find that HbA1c is significantly more important to the predictions of AutoPrognosis than blood glucose, which is consistent with our earlier experiments that showed QDiabetes Model C (which uses HbA1c) outperforms Model B (which uses blood glucose) on the UK Biobank population.

Finally, several features commonly incorporated in previous risk scores are notably missing: for example age and sex. One explanation could be that UK Biobank contains a limited age range (40–69 at enrollment), and thus the role of age could be reduced over that range. However, increasingly, younger individuals are being diagnosed with diabetes [74], which could also explain the omission of age as a key risk factor. In the case of sex, while it was once assumed that there were sex differences, diabetes is equally prevalent among men and women in most populations [75].

To illustrate debugging, we consider the development of diabetes in individuals with differing HbA1c levels. We divide the overall cohort into two approximately equal parts using the median HbA1c value of 4.69%. This equates to splitting the population into a low-normal subgroup and a high-normal and elevated subgroup [76].

We evaluated AutoPrognosis and the QDiabetes models on these two cohorts (Table 5). Despite displaying better performance across the entire dataset, QDiabetes Model C *under* performs Model A for patients in the low-normal HbA1c cohort. Conversely, AutoPrognosis performs best for both subgroups, although predicting future risk of diabetes is more challenging for low-normal HbA1c patients, in line with the other models. This could suggest that QDiabetes Model C is overly reliant on HbA1c while AutoPrognosis has more accurately captured the risk factors for low HbA1c patients.

**Table 5:** Performance of diabetes risk scores for subgroups defined by HbA1c.

Method	C-index		AUROC	
	HbA1c < 4.69%	HbA1c ≥ 4.69%	HbA1c < 4.69%	HbA1c ≥ 4.69%
QDiabetes Model A	0.771 ± 0.053	0.775 ± 0.016	0.772 ± 0.009	0.775 ± 0.023
QDiabetes Model B	0.738 ± 0.031	0.773 ± 0.010	0.738 ± 0.007	0.773 ± 0.017
QDiabetes Model C	0.735 ± 0.052	0.855 ± 0.008	0.736 ± 0.022	0.856 ± 0.004
AutoPrognosis 2.0	0.818 ± 0.047	0.889 ± 0.011	0.807 ± 0.013	0.896 ± 0.009

This raises the question of *why* AutoPrognosis is able to issue more accurate predictions for the low-normal HbA1c cohort, in particular given HbA1c is ranked as the most important feature globally (Fig 4). Table 6 shows the most important features (measured by risk effect size) for the two subgroups defined by HbA1c. While there is significant overlap, there are five unique features in the top 20 for each cohort. This type of analysis can help clinicians understand and debug the predictions of models not only for the entire population, but specific subgroups of interest.

**Table 6:** The most important features for AutoPrognosis measured by risk effect size for the two cohorts defined by median HbA1c. Features with * differ between the two cohorts. Effect size in parenthesis.

HbA1c < 4.69%	HbA1c ≥ 4.69%
*Atrial fibrillation (3.0)	*HbA1c (3.0)
Waist Size (2.8)	*Glucose (2.5)
Body Mass Index (2.7)	Weight/Height Ratio (1.5)
Weight/Height Ratio (2.7)	Waist Size (1.5)
Weight (2.7)	Body Mass Index (1.4)
Hip Size (2.2)	Weight (1.3)
Waist/Hip Ratio (1.8)	Waist/Hip Ratio (1.1)
Cystatin-c (1.6)	Hip Size (1.1)
*Kidney Disease (1.5)	Alanine Transaminase (0.87)
*Uric Acid (1.3)	Triglycerides (0.76)
Alanine Transaminase (1.1)	Gamma-Glutamyl Transferase (0.74)
*Anti-hypertensive Medication (1.1)	*HDL (0.71)
*History of Hypertension (0.99)	*C-Reactive Protein (0.70)
Triglycerides (0.97)	Cystatin-c (0.68)
Gamma-Glutamyl Transferase (0.96)	*Sex Hormone-Binding Globulin (0.67)

#### Challenge 5. Making ML models accessible and usable

**Fig 5:**
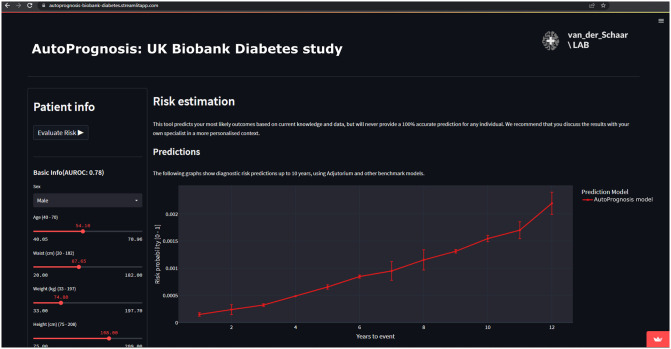
Screenshot of an example clinical demonstrator produced by AutoPrognosis.

Finally, we end our illustrative scenario with an example web-based demonstrator enabling the use of the risk model derived by AutoPrognosis. The web application can be accessed at https://autoprognosis-biobank-diabetes.streamlitapp.com/. A screenshot is provided in Fig 5.

## Discussion: Using AutoPrognosis in Healthcare and Beyond

Advances in ML algorithms harbor the potential to transform healthcare; however, major challenges continue to limit their adoption in medicine. In this work, we define these challenges and describe the first integrated, automated framework for diagnostic and prognostic modeling, AutoPrognosis 2.0, that is designed to overcome each obstacle in a way that is accessible to non-expert users, democratizing model construction, understanding, debugging, and sharing.

While AutoPrognosis seeks to address many of the algorithmic challenges of applying machine learning to clinical settings, there remains significant responsibility with the healthcare expert using AutoPrognosis to ensure appropriate study design and data curation. In particular, inappropriate use can result in inaccurate or biased results. For example, if the data used is not representative of the patient population of interest, then the model may not be applicable or accurate in real-world settings. Additionally, if the model is not adequately validated, its use could lead to a greater number of incorrect diagnoses, prognoses, or treatment recommendations than expected, which would be adverse for patient health.

In this study, we explored how AutoPrognosis could be used to construct a prognostic risk score for diabetes. The developed risk score outperformed existing approaches when evaluated on the UK Biobank cohort. However, prior to deployment in a different population, external validation should be conducted to ensure the accuracy of the risk score is not impacted by differences in patient characteristics or care.

While we have provided an illustrative example of how AutoPrognosis can be used, the key finding reported here is *not* the performance of a single illustrative model, but rather the way in which it was built. We believe AutoPrognosis 2.0 is a necessary development in the journey towards widespread adoption of ML systems in clinical practice and hope that researchers will engage with this tool. Rather than marginalizing healthcare experts, we believe AutoPrognosis places them at the center and empowers them to create new clinical tools. As part of this journey, we will continue to add new features and improve AutoPrognosis.

The adoption of AutoPrognosis and similar tools in healthcare has the potential to transform clinical decision-making and foster collaboration between ML experts and healthcare professionals. However, implementing models developed with AutoPrognosis in real-world clinical settings may present challenges, such as integration with existing medical systems. These issues are not unique to AutoPrognosis and addressing these issues will be crucial to the successful deployment of any machine learning model or other computational tools.

Finally, while the focus and motivation for AutoPrognosis is medicine, it has not escaped our notice that AutoPrognosis can be used to construct predictive models and risk scores for applications beyond healthcare.

## Supporting information

S1 Table Most important features as measured by effect size. (PDF)

S2 Table Performance of AutoPrognosis 2.0 for different subgroups. Subgroups created by splitting population on median feature value.
(PDF)

S3 Table Diabetes risk prediction results at different horizons. Mean performance reported with 95% confidence interval.
(PDF)

S4 Table Descriptive characteristics of UK Biobank cohort. (PDF)

S1 Appendix Treatment of missing values. (PDF)

S1 Fig Incidence of diabetes in the UK Biobank cohort. Proportion of cohort together with the number of individuals who have been diagnosed with diabetes for each time horizon.
(PDF)
