# Supplementary material for: AutoPrognosis 2.0: Democratizing diagnostic and prognostic modeling in healthcare with automated machine learning
Source: PLOS Digit Health. 2023 Jun 22;2(6):e0000276. doi: 10.1371/journal.pdig.0000276 (PMC10287005; doi:10.1371/journal.pdig.0000276)
Supplement: S1 Table — (PDF) [file pdig.0000276.s001.pdf]

Table S1: **Most important features as measured by effect size.**

**Feature**	**Effect size**
HbA1c	3.0
Glucose	3.0
Waist/Height Ratio	1.7
Waist Size	1.6
Body Mass Index	1.6
Weight	1.4
Hip Size	1.2
Waist/Hip Ratio	1.2
Alanine Transaminase	0.95
Triglycerides	0.88
Cystatin-c	0.86
CRP	0.83
Gamma-Glutamyl Transferase	0.82
Anti-Hypertensives	0.77
Uric Acid	0.73
HDL	0.73
Atrial Fibrillation	0.72
Sex Hormone-Binding Globulin	0.72
History of Hypertension	0.69
Chronic Kidney Disease	0.68
Urine Microalbumin	0.65
Red Blood Cell Count	0.64
Aspartate Aminotransferase	0.63
Systolic Blood Pressure	0.63
Lipid-Lowering Drugs	0.63
