# Supplementary material for: AutoPrognosis 2.0: Democratizing diagnostic and prognostic modeling in healthcare with automated machine learning
Source: PLOS Digit Health. 2023 Jun 22;2(6):e0000276. doi: 10.1371/journal.pdig.0000276 (PMC10287005; doi:10.1371/journal.pdig.0000276)
Supplement: S2 Table — Subgroups created by splitting population on median feature value. (PDF) [file pdig.0000276.s002.pdf]

Table S2: **Performance of AutoPrognosis 2.0 for different subgroups.** Subgroups created by splitting population on median feature value.

**Cohort**	**C-index**	**Brier Score**	**AUROC**
Male	0.896 ± 0.007	0.003 ± 0.000	0.897 ± 0.021
Female	0.854 ± 0.029	0.001 ± 0.000	0.848 ± 0.016
HbA1c $< 4.69\%$	0.818 ± 0.047	0.001 ± 0.000	0.807 ± 0.013
HbA1c $\geq 4.69\%$	0.889 ± 0.011	0.004 ± 0.000	0.896 ± 0.009
Age < 54.1	0.860 ± 0.026	0.002 ± 0.000	0.852 ± 0.012
Age ≥ 54.1	0.890 ± 0.013	0.003 ± 0.000	0.895 ± 0.025
Glucose < 4.94 mmol/l	0.862 ± 0.009	0.002 ± 0.000	0.870 ± 0.018
Glucose ≥ 4.94 mmol/l	0.893 ± 0.032	0.003 ± 0.000	0.896 ± 0.015
Waist/Height Ratio < 0.52	0.818 ± 0.029	0.001 ± 0.000	0.794 ± 0.036
Waist/Height Ratio ≥ 0.52	0.874 ± 0.001	0.004 ± 0.000	0.879 ± 0.012
Waist Size < 87.92 cm	0.802 ± 0.024	0.001 ± 0.000	0.796 ± 0.040
Waist Size ≥ 87.92 cm	0.878 ± 0.006	0.004 ± 0.000	0.886 ± 0.026
