# Supplementary material for: AutoPrognosis 2.0: Democratizing diagnostic and prognostic modeling in healthcare with automated machine learning
Source: PLOS Digit Health. 2023 Jun 22;2(6):e0000276. doi: 10.1371/journal.pdig.0000276 (PMC10287005; doi:10.1371/journal.pdig.0000276)
Supplement: S3 Table — Mean performance reported with 95% confidence interval. (PDF) [file pdig.0000276.s003.pdf]

Table S3: **Diabetes risk prediction results at different horizons.** Mean performance reported with 95% confidence interval.

Method	C-index ↑	Brier score ↓	AUROC ↑
3 year			
Cox PH (ADA)	0.737 ± 0.046	0.001 ± 0.000	0.726 ± 0.028
Cox PH (FINRISK)	0.753 ± 0.035	0.001 ± 0.000	0.744 ± 0.020
Cox PH (DiabetesUK)	0.773 ± 0.039	0.001 ± 0.000	0.766 ± 0.034
Cox PH (QDiabetes C)	0.880 ± 0.033	0.001 ± 0.000	0.867 ± 0.039
**AutoPrognosis 2.0**	**0.904 ± 0.035**	**0.001 ± 0.000**	**0.896 ± 0.011**
5 year			
Cox PH (ADA)	0.763 ± 0.038	0.001 ± 0.000	0.766 ± 0.029
Cox PH (FINRISK)	0.779 ± 0.032	0.001 ± 0.000	0.781 ± 0.027
Cox PH (DiabetesUK)	0.793 ± 0.032	0.001 ± 0.000	0.794 ± 0.028
Cox PH (QDiabetes C)	0.873 ± 0.020	0.001 ± 0.000	0.869 ± 0.024
**AutoPrognosis 2.0**	**0.903 ± 0.021**	**0.001 ± 0.000**	**0.902 ± 0.016**
7 year			
Cox PH (ADA)	0.765 ± 0.019	0.001 ± 0.000	0.765 ± 0.022
Cox PH (FINRISK)	0.780 ± 0.016	0.001 ± 0.000	0.779 ± 0.018
Cox PH (DiabetesUK)	0.789 ± 0.016	0.001 ± 0.000	0.787 ± 0.016
Cox PH (QDiabetes C)	0.860 ± 0.010	0.001 ± 0.000	0.859 ± 0.022
**AutoPrognosis 2.0**	**0.889 ± 0.007**	**0.001 ± 0.000**	**0.889 ± 0.028**
