# Supplementary material for: AutoPrognosis 2.0: Democratizing diagnostic and prognostic modeling in healthcare with automated machine learning
Source: PLOS Digit Health. 2023 Jun 22;2(6):e0000276. doi: 10.1371/journal.pdig.0000276 (PMC10287005; doi:10.1371/journal.pdig.0000276)
Supplement: S4 Table — (PDF) [file pdig.0000276.s004.pdf]

Table S4: **Descriptive characteristics of UK Biobank cohort.**

**Characteristic**	**Proportion**
Age	
< 50	34.7%
$50 - 54.9$	18.6%
$55 - 59.9$	18.3%
$60 - 64.9$	18.7%
≥ 65	9.8%
Missing	0.0%
Sex	
Male	43.8%
Female	56.2%
Missing	0.0%
Ethnicity	
White	94.1%
Other	5.6%
Missing	0.3%
Alcohol frequency (days per week)	
0	3.9%
$0 - 1$	21.6%
$1 - 2$	26.8%
$2 - 5$	24.9%
7	20.0%
Missing	2.8%
HbA1c	
$< 5.7\%$	90.9%
$5.7\% - 6.5\%$	1.9%
$\geq 6.5\%$	0.4%
Missing	6.8%
Glucose (mmol/l)	
< 5.6	76.8%
$5.6 - 6.9$	7.8%
≥ 6.9	1.2%
Missing	14.2%
