# Supplementary material for: AutoPrognosis 2.0: Democratizing diagnostic and prognostic modeling in healthcare with automated machine learning
Source: PLOS Digit Health. 2023 Jun 22;2(6):e0000276. doi: 10.1371/journal.pdig.0000276 (PMC10287005; doi:10.1371/journal.pdig.0000276)
Supplement: S1 Appendix — (PDF) [file pdig.0000276.s005.pdf]

S1 Appendix - Treatment of missing values

Average missingness was 4.2% and the most frequently missing variable was lipoprotein (a), which was missing for 24.4% of individuals. The only other variable exceeding 20% missingness was direct bilirubin (20.0% missing). For the following variables, we performed zero imputation: history of myocardial infarction, history of angina, history of stroke, history of ischaemic stroke, history of haemorrhagic stroke, history of hypertension, history of neoplasm, family history of CHD (parents), family history of diabetes (parents), family history of stroke (parents), smoking status, alcohol status, alcohol frequency, alcohol amount. After this step, the resulting average missingness was 3.8%. For the remaining variables, we performed imputation fives times using the HyperImpute package in AutoPrognosis.
