# Supplementary material for: AutoPrognosis 2.0: Democratizing diagnostic and prognostic modeling in healthcare with automated machine learning
Source: PLOS Digit Health. 2023 Jun 22;2(6):e0000276. doi: 10.1371/journal.pdig.0000276 (PMC10287005; doi:10.1371/journal.pdig.0000276)
Supplement: S1 Fig — Proportion of cohort together with the number of individuals who have been diagnosed with diabetes for each time horizon. (PDF) [file pdig.0000276.s006.pdf]

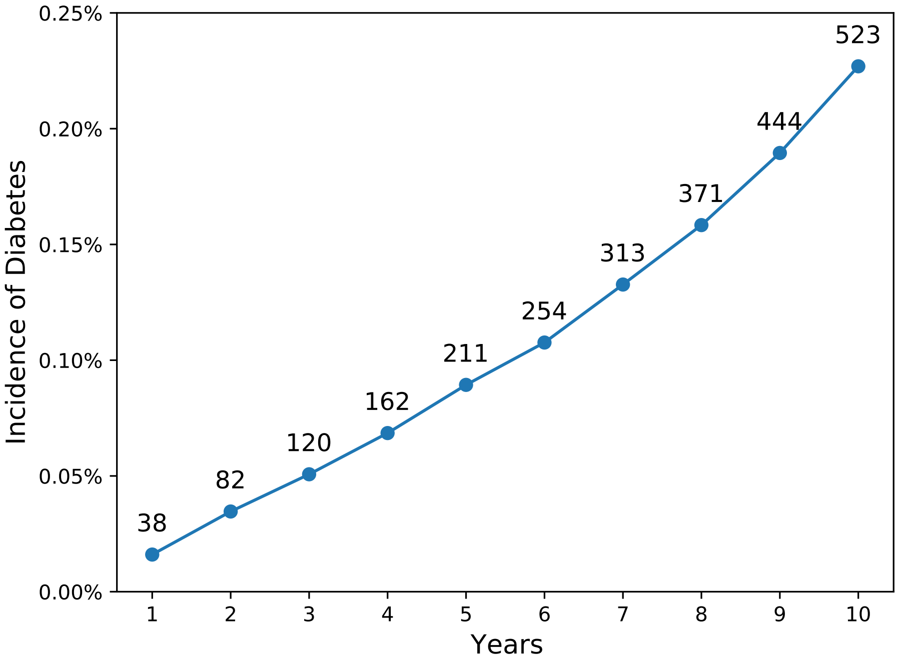

Figure S1: **Incidence of diabetes in the UK Biobank cohort.** Proportion of cohort together with the number of individuals who have been diagnosed with diabetes for each time horizon.
